# Using Bayesian Networks to Predict Long-Term Health-Related Quality of Life and Comorbidity after Bariatric Surgery: A Study Based on the Scandinavian Obesity Surgery Registry

**DOI:** 10.3390/jcm9061895

**Published:** 2020-06-17

**Authors:** Yang Cao, Mustafa Raoof, Eva Szabo, Johan Ottosson, Ingmar Näslund

**Affiliations:** 1 Clinical Epidemiology and Biostatistics, School of Medical Sciences, Örebro University, 70182 Örebro, Sweden; 2 Department of Surgery, Faculty of Medicine and Health, Örebro University, 70182 Örebro, Sweden; mustafa.raoof@regionorebrolan.se (M.R.); eva.szabo@regionorebrolan.se (E.S.); johan.ottosson@regionorebrolan.se (J.O.); ingmar.naslund@regionorebrolan.se (I.N.)

**Keywords:** machine learning-enabled decision support system, improving diagnosis accuracy, Bayesian network, bariatric surgery, health-related quality of life, comorbidity

## Abstract

Previously published literature has identified a few predictors of health-related quality of life (HRQoL) after bariatric surgery. However, performance of the predictive models was not evaluated rigorously using real world data. To find better methods for predicting prognosis in patients after bariatric surgery, we examined performance of the Bayesian networks (BN) method in predicting long-term postoperative HRQoL and compared it with the convolution neural network (CNN) and multivariable logistic regression (MLR). The patients registered in the Scandinavian Obesity Surgery Registry (SOReg) were used for the current study. In total, 6542 patients registered in the SOReg between 2008 and 2012 with complete demographic and preoperative comorbidity information, and preoperative and postoperative 5-year HROoL scores and comorbidities were included in the study. HRQoL was measured using the RAND-SF-36 and the obesity-related problems scale. Thirty-five variables were used for analyses, including 19 predictors and 16 outcome variables. The Gaussian BN (GBN), CNN, and a traditional linear regression model were used for predicting 5-year HRQoL scores, and multinomial discrete BN (DBN) and MLR were used for 5-year comorbidities. Eighty percent of the patients were randomly selected as a training dataset and 20% as a validation dataset. The GBN presented a better performance than the CNN and the linear regression model; it had smaller mean squared errors (MSEs) than those from the CNN and the linear regression model. The MSE of the summary physical scale was only 0.0196 for GBN compared to the 0.0333 seen in the CNN. The DBN showed excellent predictive ability for 5-year type 2 diabetes and dyslipidemia (area under curve (AUC) = 0.942 and 0.917, respectively), good ability for 5-year hypertension and sleep apnea syndrome (AUC = 0.891 and 0.834, respectively), and fair ability for 5-year depression (AUC = 0.750). Bayesian networks provide useful tools for predicting long-term HRQoL and comorbidities in patients after bariatric surgery. The hybrid network that may involve variables from different probability distribution families deserves investigation in the future.

## 1. Introduction

Over the past two decades, obesity has been continuously increasing worldwide, which has become a major health issue worldwide and raised public concern across the globe [1]. Severe obesity, defined as body mass index (BMI) over 35 kg/m^2^ with obesity-related comorbidities, or BMI > 40 kg/m^2^, has been associated with impaired health-related quality of life (HRQoL) and multiple comorbidities, including type 2 diabetes (T2D), hypertension, and cancer [2,3,4]. Gastric bypass and other weight-loss surgeries, known collectively as bariatric surgery, are currently considered the most effective treatment options for morbid obesity to help severe obese patients to lose excess weight and reduce potentially life-threatening risk of weight-related health problems, such as heart disease and stroke, hypertension, T2D, nonalcoholic fatty liver disease, and sleep apnea [5,6].

Based on the findings from several long-term (follow-up time ranging between 5 and 10 years) prospective studies, bariatric surgery patients’ HRQoL improved considerably after surgery and much of the initial HRQoL improvement was maintained over the long term [7]. While bariatric surgery can offer many benefits, all forms of weight-loss surgery are major procedures that can pose serious risks and side effects, including acid reflux, chronic nausea and vomiting, infection, obstruction of stomach, failure to lose weight, low blood sugar, malnutrition, and hernias, which in turn may have adverse impacts on HRQoL of the patients with morbid obesity after surgery [8,9,10].

Previously published literature has identified a few predictors of HRQoL after bariatric surgery, including baseline demographic data and depression severity score [11,12,13,14]. However, none of these studies evaluated the models’ performances or the predictors’ predictive abilities rigorously using real world data. In our previous study, we have examined the performance of the convolution neural network (CNN) for predicting 5-year HRQoL after bariatric surgery based on the available preoperative information from the Scandinavian Obesity Surgery Registry (SOReg) [15]. We found that, although the CNN is better than the traditional multivariate linear regression model at predicting long-term HRQoL after bariatric surgery, the overfitting issue is still apparent and needs to be mitigated [15]. In the two recently published studies, using the same database, we found that patients with postoperative complications had significantly less improvements in all aspects of HRQoL compared to those without any form of postoperative complication [16], and the ability of multilayer perceptron and CNN for predicting the postoperative serious complications after bariatric surgery is limited [17].

To find better methods for predicting prognosis and provide evidence for patient management after bariatric surgery, in this study, we examined the performance of the Bayesian networks (BN) method in predicting long-term postoperative HRQoL and compared it with the CNN and multivariable linear regression. At the same time, we also evaluated the performance of the BN in predicting postoperative comorbidities and compared it with multivariable logistic regression (MLR) model.

## 2. Materials and Methods

### 2.1. Subjects and Variables

The patients registered in the Scandinavian Obesity Surgery Registry (SOReg) were used for the current study. The registry was launched in 2007 and covers 98% of bariatric surgery in Sweden since 2009. It is validated regularly and shows high data quality [18,19,20,21]. In total, 6542 patients registered in the SOReg between 2008 and 2012, operated with primary Roux-en-Y gastric bypass, with complete demographic and preoperative comorbidity information; and preoperative and postoperative 5-year HROoL scores and comorbidities were included in the study. HRQoL was measured using the RAND-SF-36 [22] and the obesity-related problems (OP) scale [23] for the patients. In the present study, 35 variables were used for analyses, including 19 predictors (i.e., sex, and preoperative age, BMI, physical functioning (PF), role physical (RP), bodily pain (BP), general health (GH), vitality (VT), social functioning (SF), role emotional (RE), mental health (MH) scale, summary physical scale (PCS), summary mental scale (MCS), OP, sleep apnea syndrome (SAS), hypertension, pharmaceutically treated T2D, depression, and dyslipidemia), and 16 outcome variables (i.e., postoperative 5-year PF, RP, BP, GH, VT, SF, RE, MH, PCS, MCS, OP, SAS, hypertension, T2D, depression, and dyslipidemia). All scale scores ranged from 0 to 100, with higher scores indicating better health status, except for OP, for which low values represent good health; comorbidity variables are binary, with 1 indicating yes and 0 no.

The characteristics of the patients are shown in Table 1. Briefly, the average age and body mass index (BMI) of the patients were 42.7 years and 42.3 kg/m^2^, respectively. More than three quarters (78.8%) were female and 45% had at least one of the five comorbidities before bariatric surgery. Prevalence for all the comorbidities was reduced except for depression, and all the HRQoL scores were improved except for MCS after five years of bariatric surgery (Table 1).

The study was approved by the Regional Ethics Review Committee in Stockholm (approval number: 2013/535-31/5). The data that support the study are not publicly available because they contain information that could compromise research subjects’ privacy and confidentiality. However, the data may be available upon reasonable request and with permission of the Committee of Scandinavian Obesity Surgery Registry in Örebro, Sweden.

### 2.2. Statistical Methods

A BN is a probabilistic directed acyclic graphical model that represents a set of variables and their conditional dependencies via a directed acyclic graph (DAG). In particular, each node in the DAG represents a random variable, while the edges between the nodes represent probabilistic dependencies among the corresponding random variables. A BN takes an event that occurred and predicts the likelihood that any one of its parent nodes was the possibly contributing factor [24]. Applications of BN have multiplied in recent years, spanning such different topics as systems biology, economics, social sciences, and medical informatics [25,26].

In the current study, prediction for 5-year HRQoL scores was conducted using a Gaussian BN (GBN) because it follows or approximates a normal distribution [25]. GBN is a specially directed graphical model, which offers algorithms for prediction and inference when all variables could be defined by a Gaussian prior distribution or a Gaussian conditional distribution [27,28]. Binary predictors were transformed into continuous propensity scores using MLR before they entered the GBN [29]. Performance factors of the GBN were all compared with those from the previous CNN [15] and a traditional linear regression model.

Prediction for 5-year comorbidities was conducted using both multinomial discrete BN (DBN) and MLR, and the results from the two methods were compared. Before entering the DBN, the continuous predictors were discretized into ten categories using the information-preserving discretization method introduced by Hartemink [30]. Although at the cost of losing some information, the discretization may accommodate skewness of the variables and nonlinear relationships between them, and speed up the computation substantially [25,31].

### 2.3. Model Training and Validation

In total, 80% of the patients were randomly selected as a training dataset for learning the structure of the GBN and the DBN. When learning the structure of the networks, only a black list was used to block the edges directing from the postoperative variables to the preoperative variables, and no other constraints were used. The hill-climbing (HC) algorithm was used to learn the structure of the networks, which starts from a network with no edges, and then adds, removes, and reverses one edge at a time and picks the change that increases the network’s Bayesian information criterion score the most [25].

The remaining 20% of the patients were used as the validation dataset to evaluate the performance of the Bayesian networks, CNN, multivariable linear, and logistic regression models. Performance of the GBN was evaluated using the mean squared error (MSE) in view of the existence of zero values in the outcome variables [32]. MSE from the min-max normalized scores (between 0 and 1) was used to compare the results from the GBN and those from the previous multivariable linear regression and the CNN [33]. Performance of the DBN and MLR was evaluated using accuracy, sensitivity, specificity, and area under the receiver operating characteristic (ROC) curve. Terminology and derivations of the metrics were given in detail elsewhere [33]. A successful prediction model for comorbidities was defined as with an area under the ROC curve (AUC) greater than 0.7 [33,34,35].

### 2.4. Software and Hardware

The descriptive statistical analyses were performed using Stata 16.0 (StataCorp LLC, College Station, TX, USA). The Bayesian networks were constructed using package *bnlearn* [25,36] in software R version 3.62 (R Foundation for Statistical Computing, Vienna, Austria), and the multiple linear and logistic regression models were achieved in R as well.

All of the computation was conducted in a computer with a 64-bit Windows 7 Enterprise operation system (Service Pack 1), Intel^®^ Core TM i5-4210U CPU @ 2.40 GHz, and 16.0 GB random access memory.

## 3. Results

### 3.1. Structure of the GBN

**Figure 1:**
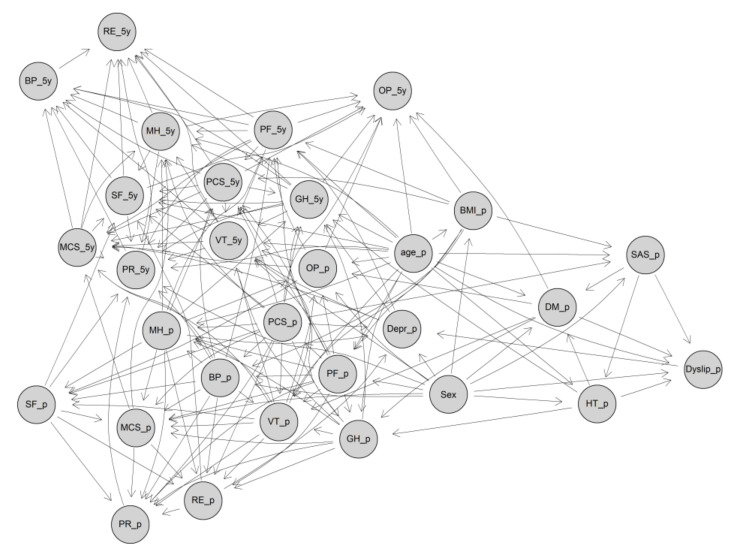
The directed acyclic graph (DAG) of the GBN for predicting postoperative 5-year HRQoL scores. DAG, directed acyclic graph; GBN Gaussian Bayesian network; PF, physical functioning; RP, role physical; BP, bodily pain; GH, general health; VT, vitality; SF, social functioning; RE, role emotional; MH, mental health; PCS, summary physical scale; MCS, summary mental scale; OP, obesity-related problems; BMI, body mass index; SAS, sleep apnea syndrome; HT, hypertension; DM, diabetes; Depr, depression; Dyslip, dyslipidemia; _p, preoperation; _5y, 5-year.

The structure of the GBN for predicting the postoperative 5-year HRQoL is shown using the DAG in Figure 1. It shows all the edges based on the HC algorithm. The DAG looks complicated and messy because it indicates all the contributors to each postoperative 5-year variable at the same time. For example, the possible direct contributors for the 5-year OP are preoperative T2D, BMI, age, OP, and PCS, and 5-year GH, PCS, SF, PF, and MH. The conditional distribution of the 5-year OP, therefore, can be presented as:

$$OP\_5y|(age_{p}=x_{1},\;BMI_{p}=x_{2},\;DM_{p}=x_{3},\;OP_{p}=x_{4},\;PCS_{p}=x_{5},\;GH_{5y}=x_{6},\;PCS_{5y}=x_{7},\;SF_{5y}=x_{8},\;PF_{5y}=x_{9},\;MH_{5y}=x_{10})\;\sim \;N\left(\beta_{0}+\beta_{1}x_{1}+\beta_{2}x_{2}+\beta_{3}x_{3}+\beta_{4}x_{4}+\beta_{5}x_{5}+\beta_{6}x_{6}+\beta_{7}x_{7}+\beta_{8}x_{8}+\beta_{9}x_{9}+\beta_{10}x_{10},\;\varepsilon^{2}\right)$$

where *N* means a normal distribution with a variance *ε*^2^.

The probability distribution above is just one of the conditional Gaussian distributions proposed by the DAG in Figure 1, and we can construct the conditional distribution for any one of the eleven postoperative 5-year HRQoL scores based on the edges pointing to them.

### 3.2. Performance of the GBN in the Validation Dataset

When the models were evaluated using the validation data that were not seen previously by the GBN, in general, the GBN presented a better performance than the CNN and the linear regression model (Table 2); all MSEs were smaller than those from the CNN and eight of eleven MSEs were smaller than those from the linear regression model (Table 2). For example, MSE of PCS was only 0.0196 for GBN compared to the 0.0333 seen in the CNN (Table 2), which means the average prediction error of the GBN accounted for less than 3% of the normalized mean of the postoperative 5-year PCS (which is 0.653). In general, the GBN could provide better prediction for postoperative 5-year HRQoL than the CNN and multivariable linear regression did.

### 3.3. Structure of the DBN

**Figure 2:**
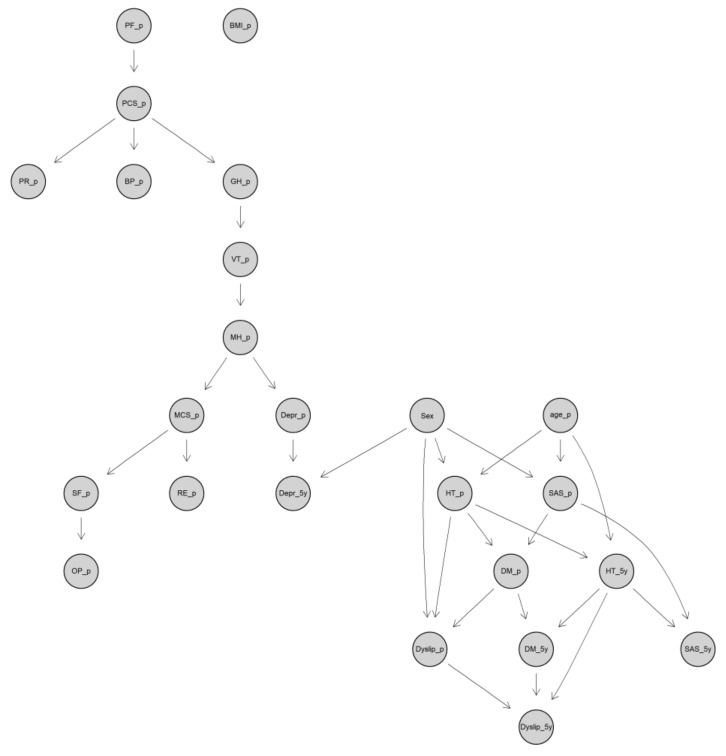
The DAG of the DBN for predicting postoperative 5-year comorbidities. DAG, directed acyclic graph; DBN, discrete Bayesian network; PF, physical functioning; RP, role physical; BP, bodily pain; GH, general health; VT, vitality; SF, social functioning; RE, role emotional; MH, mental health; PCS, summary physical scale; MCS, summary mental scale; OP, obesity-related problems; BMI, body mass index; SAS: sleep apnea syndrome; HT, hypertension; DM, diabetes; Depr, depression; Dyslip, dyslipidemia; _p, preoperation; _5y, 5-year.

The structure of DBN for predicting postoperative 5-year comorbidities is shown using the DAG in Figure 2, which is much simpler than the GBN. The comorbidities might be predicted using much less preoperative variables. For example, the conditional probability of 5-year depression (Depr_5y) depended only on sex and preoperative depression (Depr_p), which could be predicted by conditional probability tables between preoperative and postoperative depression for men or women separately. The conditional probability tables needed for men and women were estimated in a Bayesian setting in the DBN. When a comorbidity involved more predictors, such as 5-year dyslipidemia, there were more conditional probability tables to be referred to for prediction. Interestingly, preoperative BMI was not involved in any potential causal relationships in the network regarding the postoperative 5-year comorbidities (Figure 2).

### 3.4. Performance of the DBN in the Validation Dataset

**Figure 3:**
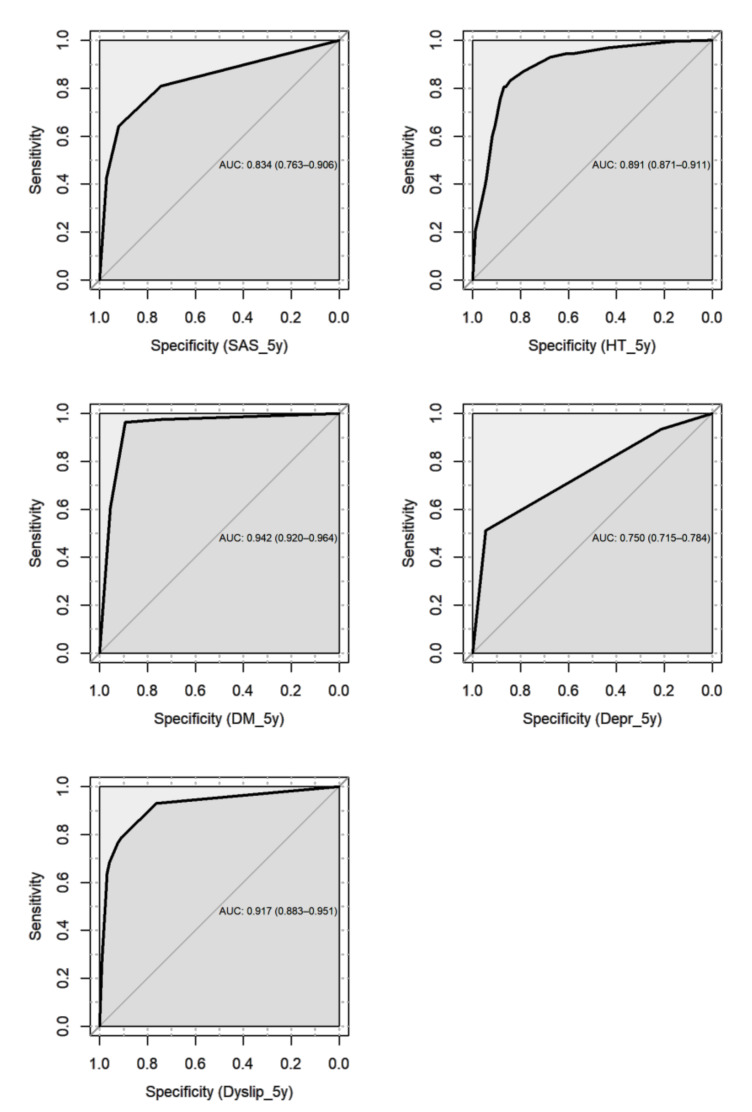
Receiver operating characteristic (ROC) curve of the discrete Bayesian network (DBN) for predicting 5-year comorbidity after bariatric surgery.

The DBN showed excellent predictive ability for 5-year T2D and dyslipidemia (AUC = 0.942 and 0.917, respectively), good ability for 5-year hypertension and SAS (AUC = 0.891 and 0.834, respectively), and fair ability for 5-year depression (AUC = 0.750) (Figure 3).

Compared with the results from the MLR, the DBN presented significant improvement in predicting 5-year comorbidities. All the AUCs from the DBN were larger than those from the MLR, and the differences were statistically significant (*p* < 0.05), except for SAS (Table 3). The sensitivity and specificity of the DBN in predicting postoperative 5-year T2D could be as high as 0.96 and 0.89, in contrast to the 0.78 and 0.68 of the MLR, respectively (Table 3).

## 4. Discussion

In this study, we explored application of Bayesian networks for predicting long-term outcomes after bariatric surgery in a national registry. They showed promising predictive ability for both continuous and binary outcomes. For predicting the postoperative 5-year HRQoL, the GBN had smaller MSEs than those seen from the CNN for all scores and from the traditional multivariable linear regression for most scores. The most accurate predictions from the GBN were seen for PCS, and followed by PF and MCS; average prediction errors were lower than 3%, 4%, and 6% of their normalized means, respectively. For predicting the postoperative 5-year comorbidity, the DBN showed statistically significantly better performance compared with the MLR. It showed good and even excellent predictive ability for four of the five comorbidities, with an AUC as high as 0.942 in postoperative T2D.

Bayesian networks use Bayesian inference to model conditional dependence, and therefore causation, via a DAG. They are ideal for taking an event that occurred and predicting the likelihood that any one of several possible known causes was the contributing factor. Experience has shown that Bayesian networks and associated methods are geared to reasoning with uncertainty in a way closely resembling physicians [37,38,39]. Physicians who aim to develop computer-assisted systems for making clinical decisions are frequently confronted by the complexity and uncertainty in the models and prediction. In many cases, the situation is even worse, as many of the processes in medicine are only partly known [38]. During the past decade, Bayesian networks have become important tools for building decision-support systems in medical sciences and are now steadily becoming mainstream in some areas [40]. However, we should notice that DAGs are not designed to capture cyclic patterns, such as depression causing increased BMI [41]. Potential cyclic causal relationships may be explored using cyclic structural equation models [42] or Markov networks [43].

Many methods have been applied to predict the outcomes in patients after bariatric surgery, including stepwise multivariable linear regression [44,45], MLR [46], and machine learning methods such as the decision tree [47] and CNN [15,33]. Although an intelligent decision-making support system involving Bayesian networks has been reported for the nutrition diagnosis of bariatric surgery patients [48], according to our literature search, there is no study that has used the method for predicting outcomes after bariatric surgery. In our previous study, we illustrated that CNN might be a useful tool to predict long-term HRQoL after bariatric surgery; however, its overfitting on external validation dataset was still noticeable. To further mitigate the overfitting issue commonly seen in the machine learning field, we explored the application and performance of Bayesian networks in the current study and achieved desired results.

A significant advantage of the study in clinical sense is that it provides a solution with which to predict outcomes as far as 5 years after bariatric surgery. To give realistic and relevant information about the long-term prognosis of bariatric surgery is currently challenging. This type of knowledge can be used in clinical practice when it comes to giving scientifically-based preoperative information to patients considering the surgery. The knowledge can also be helpful in giving scientifically-based information to policy makers in health care to explain the expected positive effects of bariatric surgery. This information can also be used to customize the follow-ups of the individual patients. However, we would also note that this kind of prediction should not be used to exclude individual patients, who otherwise fulfil criteria for surgery, from having an operation. Meanwhile, while limited by the relatively small sample size compared to those usually recommended in statistical learning studies, it would be premature to use the models presented in the study in clinical decision-making right now.

There are several advantages in Bayesian networks. First, commonly used methods in epidemiological studies such as logistic regression and related methods do not take account of causal relationships that may exist between the covariates. Causal relationships between some of the risk factors may be already known, or may be regarded as plausible on biological grounds [49,50]. However, such information was incorporated into our BN models to reveal the potential relationships between the health or disease status and the associated risk factors [51]. Second, high correlation among predictors has long been an annoyance in regression analysis. The crux of the problem is that the linear regression model assumes each predictor has an independent effect on the response that can be encapsulated in the predictor’s regression coefficient. As opposed to creating problems of multicollinearity, the associations between candidate predictor variables are naturally accounted for when defining a BN’s conditional probability distributions. The HC algorithm used in the study may search a structure starting from either an empty, full, or possibly random DAG, or an initial DAG chosen according to existing knowledge. The main loop then consists of attempting every possible single-edge addition, removal, or reversal relative to the current candidate network. The change that increases the score the most then becomes the next candidate. The process iterates until a change in a single-edge no longer increases the score. By gradually taking into account the relationships between the variables, the problem of multicollinearity, therefore, can be reduced in a BN analysis [52]. Third, the DAG proposed by the BN method captures the dependence structure of multiple variables, and used appropriately, allows more robust conclusions about the direction of causation. BN analysis revealed a richer structure of relationships than could be inferred using the traditional multivariable regression methods, such as logistic regression, and highlight a potential pathway unseen previously for further investigation [53]. Fourth, compared with the deep learning method CNN used in our previous study for predicting HRQoL scores, the GBN provided much faster computing, better performance, and interpretable results. Finding the final DAG with the HC algorithm using 35 variables and 6542 observations only took 2 min in GBN analysis, in contrast to about 10 min in CNN analysis [33]. Except for the output HRQoL scores, the contributions of and relationships between the variables could not be explained or were hard to explain in the CNN analysis. In contrast, the GBN showed us all the potential causal relationships between the variables and estimated the strength of the relationships using liner regression coefficients.

However, there are limitations in our study. Our dataset includes both continuous and binary variables. To reduce the complexity of the networks and computing time, we converted the binary variables to continuous propensity scores for the GBN analysis, and discretized the HRQoL scores to categorical variables for the DBN analysis, which may involve tortuous information or lose some information in the analyses. A better solution would be a hybrid BN with use of Markov chain Monte Carlo techniques [25]. Although limited by the software packages available and adopting the compromising methods so far, we would like to explore the hybrid BN in the future and see whether it could improve the performance of prediction further. Besides, even though HRQoL and comorbidities are of importance, we have not tested hard endpoints, such as survival, heart attack, stroke, and cancer, which warrants a subsequent study when more detailed data are available. We should also notice that this study only included patients from Roux-en-Y gastric bypass, since this was almost the only operation method used in Sweden during the study period. Whether the results could be applied to other methods, such as sleeve gastrectomy, is not known yet. However, we will be able to investigate this in the future, since SOReg has contained a large number of sleeve gastrectomy patients in recent years. Besides, there are many more females than males in the database (80% vs. 20%). The generalizability of the BN models might be limited by the gender imbalance. Meanwhile, the menopausal transition can be an important factor related to HRQol in women [54]. In view of the average age with a wide standard deviation at 5 years after surgery, which is right around the menopause of women, this issue deserves clarification and assessment by incorporating with the menopause information in women. Therefore, the applicability and validity of the models need be further explored using a larger representative dataset with more covariates and longer follow-up.

## 5. Conclusions

Bayesian networks provide useful tools for predicting long-term HRQoL and comorbidities in patients after bariatric surgery, based on their preoperative health and disease status. The GBN and DBN used in our study outperformed the deep learning method CNN and multivariable logistic regression. However, the hybrid network that may involve variables from different probability distribution families deserves investigation in the future.

## Figures and Tables

**Table 1:** Characteristics of the patients (*n* = 6542) included in the study, mean ± SD or *n* (%).

	Preoperative	5-Year Postoperative
Age (year)	42.7 ± 11.0	47.7 ± 11.0
BMI (kg/m^2^)	42.3 ± 5.2	30.3 ± 5.2
Female	5154 (78.8%)	5154 (78.8%)
SAS	668 (10.2%)	188 (2.9%)
Hypertension	1817 (27.8%))	1420 (21.7%)
T2D	973 (14.9%)	452 (6.9%)
Depression	855 (13.1%)	1162 (17.8%)
Dyslipidemia	732 (11.2%)	429 (6.6%)
PF	61.7 ± 21.9	84.2 ± 20.7
RP	60.3 ± 38.9	77.9 ± 36.5
BP	56.0 ± 26.9	65.2 ± 30.7
GH	58.2 ± 21.4	68.1 ± 24.7
VT	47.4 ± 23.0	54.5 ± 26.9
SF	74.9 ± 26.1	79.6 ± 26.4
RE	76.0 ± 36.2	76.9 ± 37.8
MH	71.5 ± 19.4	72.0 ± 23.0
PCS	38.3 ± 10.7	47.6 ± 11.1
MCS	46.8 ± 11.7	44.6 ± 13.8
OP	61.0 ± 26.4	25.6 ± 27.4

SD, standard deviation; BMI, body mass index; SAS, sleep apnea syndrome; T2D, type 2 diabetes; PF, physical functioning; RP, role-physical; BP, bodily pain; GH, general health; VT, vitality; SF, social functioning; RE, role-emotional; MH, mental health; PCS, summary physical scale; MCS, summary mental scale; OP, obesity-related problems.

**Table 2:** Mean squared errors of the GBN, the CNN, and the multivariable linear regression model.

HRQoL Scores	GBN	CNN	Linear Regression
PF	0.0335	0.0350	0.0343
RP	0.1166	0.1324	0.1211
BP	0.0813	0.0898	0.0772
GH	0.0499	0.0618	0.0508
VT	0.0590	0.0914	0.0625
SF	0.0599	0.0995	0.0588
RE	0.1230	0.2118	0.1269
MH	0.0436	0.0807	0.0416
PCS	0.0196	0.0333	0.0219
MCS	0.0356	0.0584	0.0305
OP	0.0597	0.0750	0.0608

GBN, Gaussian Bayesian network; CNN, convolutional neural network; PF, physical functioning; RP, role physical; BP, bodily pain; GH, general health; VT, vitality; SF, social functioning; RE, role emotional; MH, mental health; PCS, summary physical scale; MCS, summary mental scale; OP, obesity-related problems.

**Table 3:** Performance metrics of the DBN and MLR model for predicting the 5-year comorbidities.

Comorbidity	DBN				MLR			
	Sen	Spe	Acc	AUC (95% CI)	Sen	Spe	Acc	AUC (95% CI)
SAS	0.64	0.92	0.91	0.83 (0.76, 0.91)	0.90	0.73	0.73	0.90 (0.86, 0.94)
Hypertension	0.83	0.83	0.84	0.89 (0.87, 0.91)	0.73	0.67	0.68	0.76 (0.73, 0.79)
T2D	0.96	0.89	0.90	0.94 (0.92, 0.96)	0.78	0.68	0.69	0.76 (0.72, 0.81)
Depression	0.51	0.95	0.87	0.75 (0.72, 0.78)	0.66	0.55	0.57	0.61 (0.67, 0.65)
Dyslipidemia	0.78	0.91	0.90	0.92 (0.88, 0.95)	0.76	0.67	0.68	0.77 (0.74, 0.82)

DBN, discrete Bayesian network; MLR, multivariable logistic regression; Sen, sensitivity; Spe, specificity; Acc, accuracy; AUC, area under the ROC curve; CI, confidence interval; SAS: sleep apnea syndrome; T2D, type 2 diabetes.
